# Strengthening of Aluminum Wires Treated with A206/Alumina Nanocomposites

**DOI:** 10.3390/ma11030413

**Published:** 2018-03-10

**Authors:** David Florián-Algarín, Raúl Marrero, Xiaochun Li, Hongseok Choi, Oscar Marcelo Suárez

**Affiliations:** 1 Department of Civil Engineering, University of Puerto Rico-Mayagüez, Mayagüez, PR 00681, USA david.florian@upr.edu; 2 Department of Civil and Environmental Engineering, Northwestern University, Evanston, IL 60208, USA; raulmarrero2015@u.nortwestern.edu; 3 Department of Mechanical and Aerospace Engineering, University of California-Los Angeles, Los Angeles, CA 90095-1597, USA; xcli@seas.ucla.edu; 4 Department of Mechanical Engineering, Clemson University, Clemson, SC 29634, USA; hongc@clemson.edu; 5 Department of Engineering Science and Materials, University of Puerto Rico-Mayagüez, Mayagüez, PR 00681, USA

**Keywords:** aluminum nanocomposites, aluminum welding, TIG fillers, electrical conductivity, wire fabrication

## Abstract

This study sought to characterize aluminum nanocomposite wires that were fabricated through a cold-rolling process, having potential applications in TIG (tungsten inert gas) welding of aluminum. A206 (Al-4.5Cu-0.25Mg) master nanocomposites with 5 wt % γAl_2_O_3_ nanoparticles were first manufactured through a hybrid process combining semi-solid mixing and ultrasonic processing. A206/1 wt % γAl_2_O_3_ nanocomposites were fabricated by diluting the prepared master nanocomposites with a monolithic A206 alloy, which was then added to a pure aluminum melt. The fabricated Al–γAl_2_O_3_ nanocomposite billet was cold-rolled to produce an Al nanocomposite wire with a 1 mm diameter and a transverse area reduction of 96%. Containing different levels of nanocomposites, the fabricated samples were mechanically and electrically characterized. The results demonstrate a significantly higher strength of the aluminum wires with the nanocomposite addition. Further, the addition of alumina nanoparticles affected the wires’ electrical conductivity compared with that of pure aluminum and aluminum–copper alloys. The overall properties of the new material demonstrate that these wires could be an appealing alternative for fillers intended for aluminum welding.

## 1. Introduction

Welding, as a critical technique to join structural parts, requires a reliable filling material [1]. In particular, welding Al alloys with a proper filling material has become more important in setting up lightweight structures, such as those in aerospace applications [1,2,3,4,5]. Therefore, tuning the mechanical and thermal properties of the filler material is of vital importance for the welding quality. In effect, high strength prevents failure of the weld, eventually enhancing the performance of the final product. In terms of the weld thermal properties, the melting temperature of a welding material should be lower than that of the parts to be joined. One needs to recall that as the metal strength levels increase, the melting point is normally higher. This means that the balance between the strength and the melting point of the welding materials is of great importance.

Metal matrix composites bearing nanostructured components (nanoparticles, nanofibers, nanotubes, or graphene) embedded in a light metallic matrix have been considered for a number of applications due to their low density, high strength, and high elastic modulus, among other properties. For instance, ceramic nanoparticles, such as silicon nitride (Si_3_N_4_), silicon carbide (SiC), zirconia (ZrO_2_), and alumina (Al_2_O_3_), are reinforcements utilized in nanocomposites because of their high temperature strength, high wear resistance, chemical stability, and large elastic modulus [6,7]. Among those ceramic nanoparticles, γAl_2_O_3_ is an effective reinforcement for aluminum due to its high hardness, mechanical strength, and good thermal shock resistance, allowing for metal matrix composites with high mechanical strength [8,9]. In addition, most engineering alumina parts come from γAl_2_O_3_ sintering. The ensuing phase development is then: γAl_2_O_3_→δAl_2_O_3_→θAl_2_O_3_→αAl_2_O_3_ [10,11]. Therefore, the cost of the δ, θ, and α phases is higher compared to the γ polymorph. Synthesized as nanoparticles, γAl_2_O_3_ can then be added as nanodispersoids to formulate an aluminum matrix nanocomposite, i.e., a nanoreinforced Al alloy.

It is apparent that effective incorporation of the ceramic particles into the aluminum (or aluminum alloy) matrix is paramount to obtaining a homogeneous and sound composite, free of pores or a weak ceramic/matrix interface (for load transfer purposes). The methods described above are effective but most of the time it is costly to attain a strong reinforcement/matrix interface. In this respect, reactive mixing could be an alternative to form a strong ceramic/metal interface, although at a price [12]. Moreover, as the size of the reinforcements becomes smaller (nanocomposites), the potential for agglomeration becomes higher. Thus, we propose the use of an aluminum alloy with well-embedded nanoparticles as a master material to inoculate an aluminum melt so as to formulate an Al matrix composite with superior strength. Such is the target of the present research, which evaluates the fabrication of a nanocomposite containing alumina nanoparticles (incorporated as part of an Al–4.5Cu–0.25Mg–1% γAl_2_O_3_ composite). The effect of its addition on the physical properties of aluminum wires, namely electrical resistivity, density, and melting point, are also studied, along with the stiffness and strength of the reinforced material. Our ultimate purpose is to produce a filler material for potential use in aluminum welding via a TIG (tungsten inert gas) method; this new material could be suitable for aerospace applications, specifically for structural joints.

## 2. Experimental Procedure

To produce the wires, three stages were necessary. The first stage was the manufacture of a master nanocomposite bearing an A206 alloy matrix [13] and containing γAl_2_O_3_ nanoparticles. This master composite was used then to inoculate an aluminum melt to attain a metal with a smaller amount of nanoparticles. The last stage was the wire drawing and subsequent characterization of its microstructure, electrical resistivity, thermal behavior, and mechanical strength.

### 2.1. Fabrication of a Master Al Matrix Nanocomposite

Table 1 shows the chemical composition of the A206 alloy used as the master composite matrix. An initial melt with 27 kg of A206 and 1 wt % of γAl_2_O_3_ was prepared using semi-solid mixing and ultrasonic processing. To enhance the semi-solid mixing of the nanoparticles, we used an axial impeller with a 25.4 mm diameter placed at a third of the total height of the crucible containing the A206 slurry. As the impeller rotated at 500 rpm, it formed a vortex into which 1.6 g of γAl_2_O_3_ nanoparticles (with an average 50 nm size) was added. Thereupon, we raised the impeller angular velocity to 1200 rpm and set the mixing time at 40 s. This feeding and mixing step was repeated until 5 wt % of nanoparticles had been added to the melt.

After the semi-solid mixing at 630 °C, we raised the temperature (700 °C) to enhance the distribution and dispersion of the nanoparticles via ultrasonic processing. To this purpose, the tip of a niobium (C-103) ultrasonic probe with a diameter of 12.7 mm and a length of 92 mm was inserted about 6.35 mm into the melt. A 20 kHz ultrasonic vibration bearing an amplitude (peak-to-peak) of 60 µm was generated by a transducer (Sonicator 3000, Misonix Inc., Farmingdale, NY, USA) and applied to the melt for 15 min. Then, the melt was heated to 740 °C for pouring into low-carbon steel rectangular molds preheated at 350 °C to obtain master nanocomposite ingots weighing 500 g. A similar ultrasonic processing was used in prior research where more details are provided [14]. 

The prepared A206/5 wt % γAl_2_O_3_ master nanocomposites were diluted with as-received A206 alloy to fabricate A206/1 wt % γAl_2_O_3_ nanocomposite castings. The scale-up ultrasonic processing system and a grade 5 titanium alloy ultrasonic probe were used for 30 min to further disperse and distribute the nanoparticles. After that, the melt was cast into permanent and sand molds. Standard A206 aluminum alloy is known for its hot tearing trend, which can be counteracted by adding γAl_2_O_3_ nanoparticles as proven in prior research [15,16]. Because the parent ingots needed to be tested for integrity in order to produce convenient inoculation shapes (i.e., wires), after pouring the melt, the hot tearing susceptibility of the nanocomposites was assessed by a constrained rod casting (CRC) with a steel mold, similar to those used in prior work [17].

### 2.2. Wire Fabrication and Characterization

This research segment involved the production of a nanocomposite bearing a matrix made of aluminum with 4.5 wt % Cu and 0.25 wt % Mg. We used 1 wt % γAl_2_O_3_ nanoparticles as reinforcement. To this purpose, pure aluminum (99.5%) was melted at 760 °C and the A206/1 wt % γAl_2_O_3_ nanocomposite was added to the mechanically stirred melt.

We poured the treated melt into a cylindrical mold to produce 6 mm diameter ingots. These underwent full annealing at 400 °C for 5 h to allow cold-rolling to obtain 2.6 mm diameter wires with a cross-sectional area reduction of 81%. Full annealing for 5 h at 400 °C permitted further cold rolling to reduce the wire diameter to 1.4 mm. Finally, another full annealing at 400 °C for 5 h allowed for a 1 mm wire diameter, which was used in prior research [18,19]. The standard tensile tests at room temperature took place in a low force universal testing machine, Instron 5944, following the ASTMB557-06 standard [20]. The fractured wire surfaces were observed in a JEOL SEM-6390 scanning electron microscope (SEM, Tokyo, Japan). We cut, ground, and polished the wires to observe their microstructure (wires at different stages of the manufacturing process) in a Nikon Epiphot 200 optical microscope (Tokyo, Japan).

A four-point probe technique allowed the electrical resistivity of the wires to be measured at different temperatures [21]. The apparatus measured the voltage drop between two probing points as different levels of current were applied to the wire via two electrodes. Then, by measuring the sample geometry, we computed the bulk material conductivity. The wires’ electrical conductivity measurements were carried out at temperatures ranging from 0 to 100 °C to simulate some operating conditions. Finally, we measured the electrical conductivity as a percent of IACS, as the International Annealed Copper Standard (IACS) indicates [22]. Table 1 and Table 2 present the target chemical composition of the A206 alloy and the aluminum wires treated with the A206/1 wt % γAl_2_O_3_ nanocomposite. Four specimens of each composition were manufactured.

Due to the low Mn, Mg, and Ti levels in the wires, we opted to disregard their effects on the properties of the wires to be studied. Conversely, we were interested in assessing how different amounts of γAl_2_O_3_ and Cu could influence those properties. To differentiate between the effects of γAl_2_O_3_ and Cu in mechanical and electrical responses, aluminum–copper wires were fabricated bearing 0.562 wt % Cu, 1.125 wt % Cu, 1.687 wt % Cu, and 2.250 wt % Cu, using the same procedure, and an Al-33 wt % Cu (eutectic composition) master alloy as the charge material. 

## 3. Results

### 3.1. Master A206/γAl_2_O_3_ Composite Characterization

**Figure 1:**
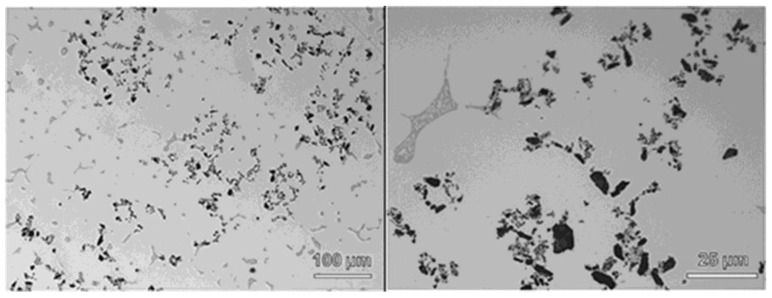
Optical micrographs of the A206/5 wt % γAl_2_O_3_ master nanocomposite alloy obtained at two magnifications (Reproduced with permission of the ASME from [16]).

**Figure 2:**
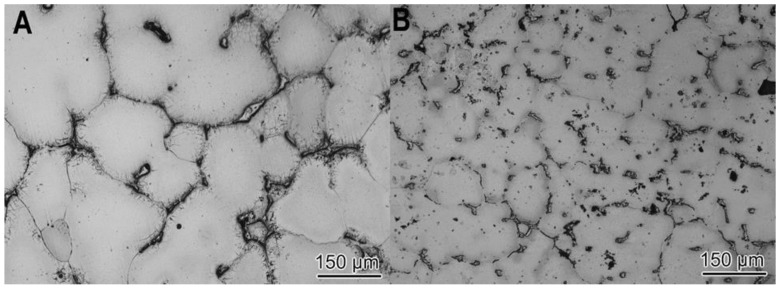
Optical micrographs of the alloy. (**A**) A206 aluminum alloy; (**B**) A206/1 wt % γAl_2_O_3_ master nanocomposite alloy.

Figure 1 shows two optical micrographs of the fabricated master nanocomposite alloy, obtained at different magnifications [16]. The images demonstrate that the γAl_2_O_3_ nanoparticles were well incorporated into the matrix and nearby the θ (Al_2_Cu)/aluminum eutectic regions. Although it is also observed that the γAl_2_O_3_ nanoparticle clusters ended up pushed into interdendritic regions, i.e., last regions to solidify, those small agglomerates would be further dispersed and distributed during the subsequent scale-up ultrasonic processing, as can be seen in Figure 2. It should be noted that 1 wt % of γAl_2_O_3_ nanoparticles, which would serve as reinforcements in the master nanocomposite alloy, were successfully incorporated into the matrix through the hybrid mixing method.

**Figure 3:**
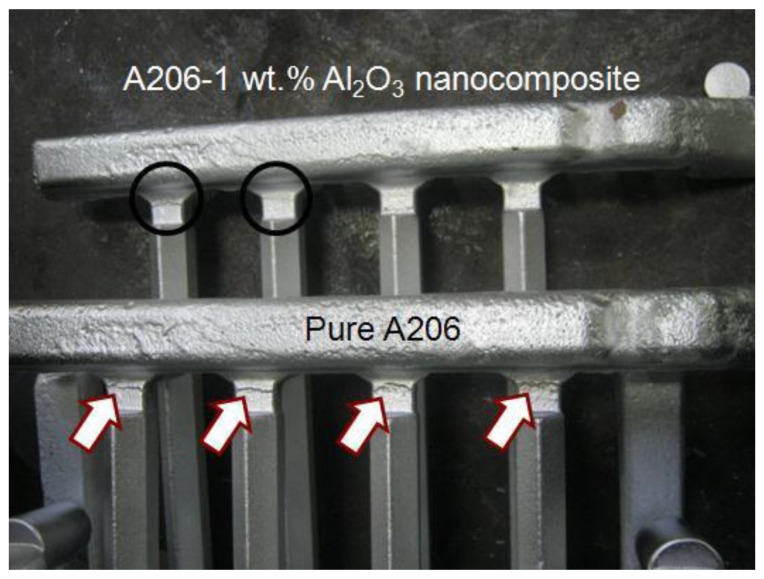
Comparison of pure A206 and A206/1 wt % γAl_2_O_3_ nanocomposite.

This is an important starting point since the nanocomposite was intended to be used as the γAl_2_O_3_ nanoparticle carrier for the posterior inoculation of the aluminum melt. The small agglomeration was, therefore, a non-issue since the clusters were uniformly distributed throughout the parent material. The hot tearing susceptibility of the nanocomposites was compared with that of pure A206 alloy. As shown in Figure 3, the hot tearing resistance of the A206/1 wt % γAl_2_O_3_ nanocomposite was significantly better than that of the pure A206 alloy. The pure A206 casting evinced severe tears (marked with arrows) all around the part; conversely, the nanocomposite had only small tears (marked with circles) and partially around the part. This ensured the integrity of the ingots and warranted the soundness of the wires for plastic deformation in an ensuing stage of the processing.

### 3.2. Wire Characterization

We deem it important to underscore that a rigorous characterization of the reinforced wires paves the way to their potential applications in the aforementioned industries. Therefore, a purely mechanical study would not suffice to evaluate the compliance of this new material with industrial specifications.

#### 3.2.1. Tensile Test Results

**Figure 4:**
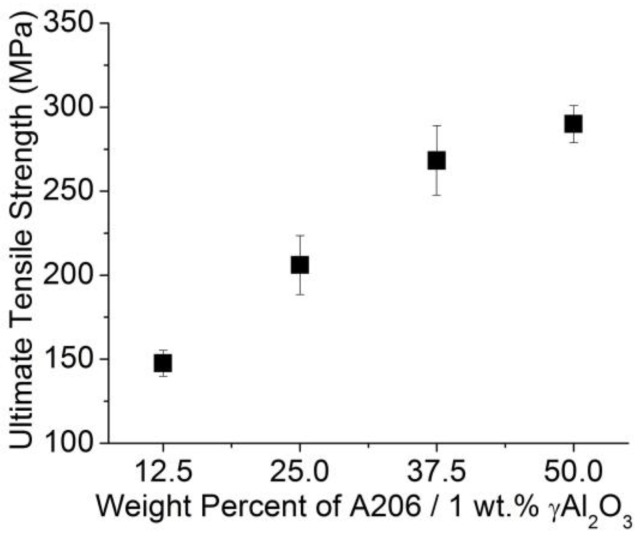
Average ultimate tensile strength of aluminum wire samples as a function of the amount of A206/1 wt % γAl_2_O_3_ added.

**Figure 5:**
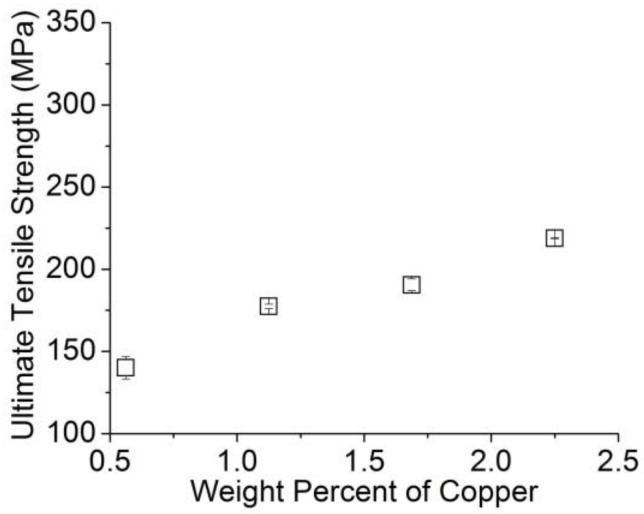
Average ultimate tensile strength of aluminum wire samples as a function of the amount of copper added.

Figure 4 and Figure 5 present the ultimate tensile strength (UTS) results; the strain rate was 1 mm/min and the initial length of the wires was 250 mm, following the ASTMB557-06 standard. Clearly, the UTS increased as the amount of A206/1 wt % γAl_2_O_3_ (i.e., the amount of nanoparticles) and copper increased. Naturally, when nanoparticles are present, the increase in the UTS is attributed to Orowan strengthening mechanisms. The isotropic and uniform distribution of the nanoparticles present in the said matrix provided effective obstacles against dislocation slippage upon plastic deformation. Similar trends have been well documented when SiC, Al_2_O_3_, Y_2_O_3_, SiO_2_, and carbon nanotube particles are embedded in pure magnesium and Mg alloys as matrix material [23]. In aluminum alloys, to increase the mechanical strength, one can use solid solution, cold working with or without heat treatment, as well as second phase precipitates and nanodispersions or nanoparticles, as in our case [24].

#### 3.2.2. Electrical Conductivity Measurements

**Figure 6:**
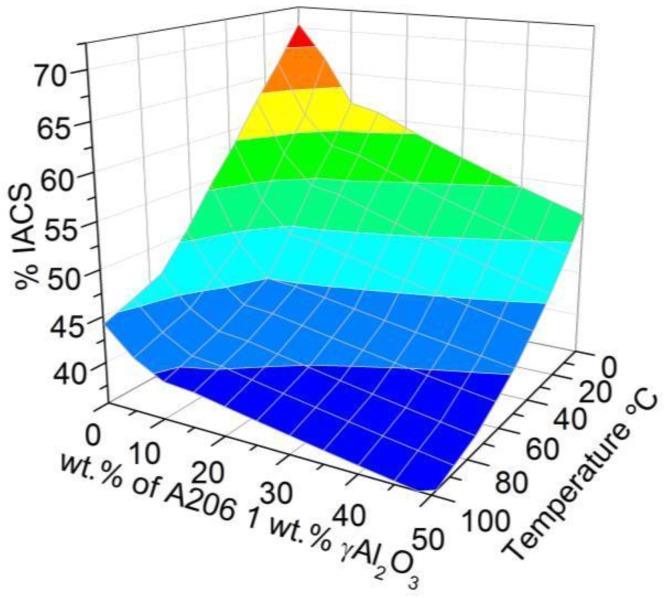
Effect of the amount of A206/1 wt % γAl_2_O_3_ nanocomposite added and the temperature on the electrical conductivity of aluminum wires (measured as a percent of International Annealed Copper Standard (IACS)).

**Figure 7:**
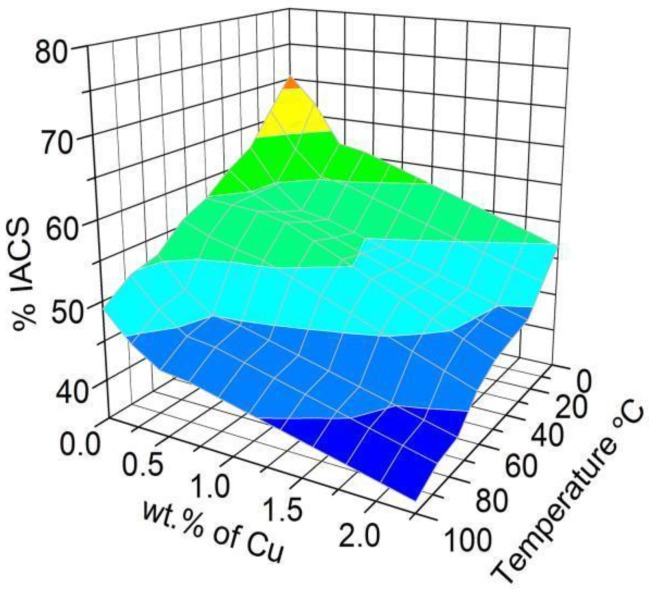
Effect of the amount of copper added and the temperature on the electrical conductivity of aluminum wires (measured as a percent of IACS).

The wires treated with the A206/1 wt % γAl_2_O_3_ nanocomposite and copper displayed lower electrical conductivity than aluminum at 25 °C, for alumina and copper concentrations ranging from 0.125 to 0.5 wt % and 0.562 to 2.250 wt %, respectively, as observed in Figure 6 and Figure 7.

#### 3.2.3. Bending (Looping) Properties

**Figure 8:**
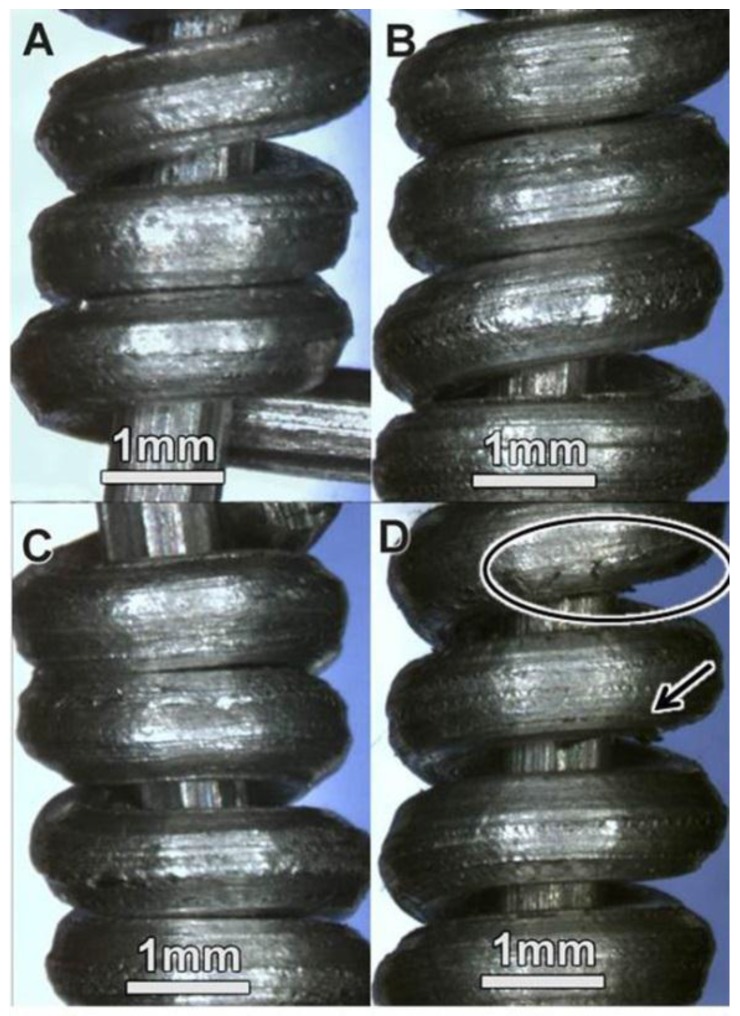
Bending (looping) test of aluminum wires treated with different weight percentage of A206/1 wt % γAl_2_O_3_ nanocomposite: (**A**) 12.5; (**B**) 25.0; (**C**) 37.5; and (**D**) 50.0.

To test the ability of a wire to be spooled without fracturing, the ASTM B 230/B 230M-07 standard can be used. This recommends looping the wire around its own diameter or about a mandrel [25]. This procedure reveals whether the wire is ductile enough to form a spool. As Figure 8 (A through C) demonstrates, the aluminum wires treated with A206/1 wt % γAl_2_O_3_ nanocomposites to 12.5, 25.0, and 37.5 weight percent did not display any fissure or crack. For the aluminum wire treated with 50.0 wt % of A206/1 wt % γAl_2_O_3_, two fractures became visible (Figure 8D). Thus, upon analyzing the bending properties and the macrograph of the wire fractures, one can conclude that increasing the A206/1 wt % γAl_2_O_3_ content of the samples caused ductility loss. This is an important finding that needs to be considered when manufacturing these types of wires.

#### 3.2.4. Density of Wires

**Figure 9:**
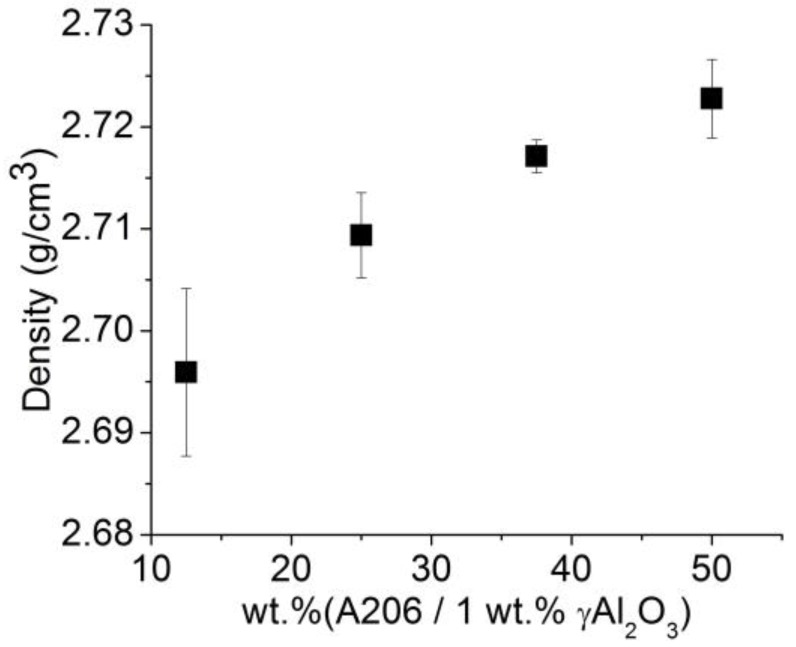
Measured density of aluminum wire samples as a function of the amount of A206/1 wt % γAl_2_O_3_ added.

In Figure 9, it is apparent that the wire density increased slightly (no more than 1%) with the amount of A206/1 wt % γAl_2_O_3_ added to the melt. As expected, the alumina and copper content did affect the density of the wires; this is because the density of the alumina and copper were ~47% and ~231% higher than aluminum, respectively [24].

#### 3.2.5. Fractographic Study of Wires

**Figure 10:**
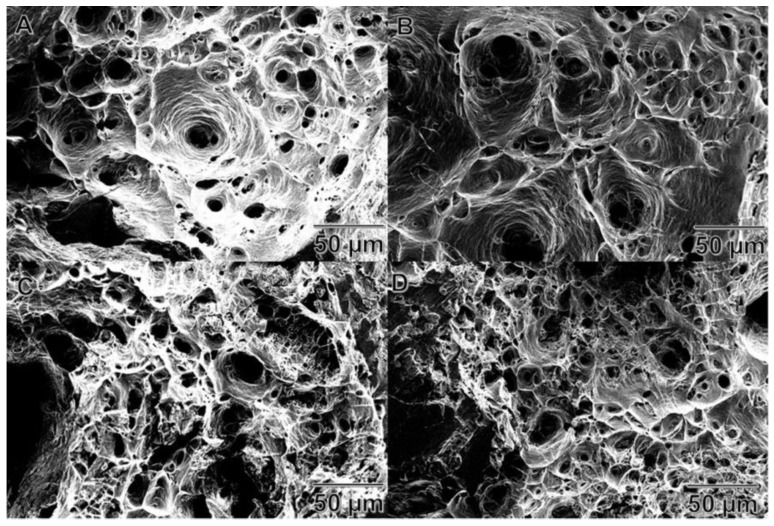
Secondary electron images of the tensile fractures of aluminum wires: (**A**) wires with 12.5 wt % (A206/1 wt % γAl_2_O_3_); (**B**) wires with 25.0 wt % (A206/1 wt % γAl_2_O_3_); (**C**) wires with 37.5 wt % (A206/1 wt % γAl_2_O_3_); and (**D**) wires with 50.0 wt % (A206/1 wt % γAl_2_O_3_).

**Figure 11:**
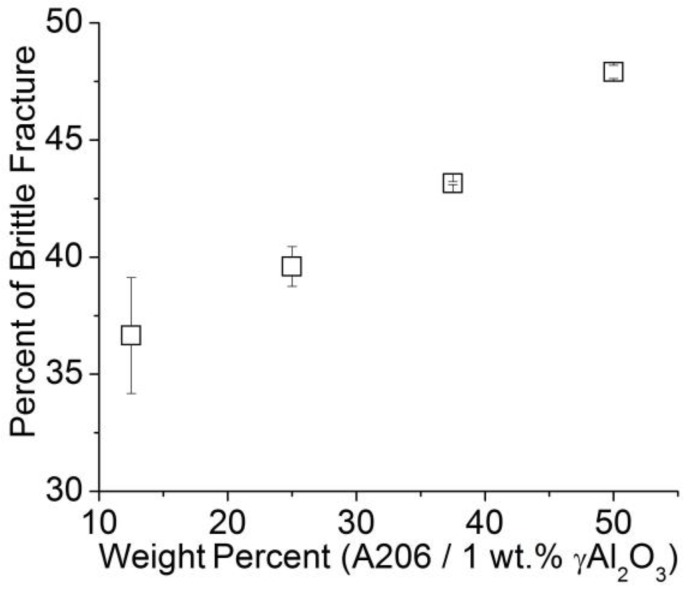
Percent of brittle fracture area in aluminum wires.

Figure 10 shows the scanning electron images of the fractures after the tensile tests of the different wires studied. The public domain ImageJ image analysis software was used to measure the area percent of brittle and ductile fractures. The original images were binarized (black and white) where the brittle area was presented as black and the ductile as white. Then, on the calibrated black and white image, the percentage of brittle and ductile fractures was measured. This procedure was developed and used in previous work [26]. Brittle area measurements (in percent) are shown in Figure 11 where a higher amount of A206/1 wt % γAl_2_O_3_ added yielded more brittleness of the treated wires.

#### 3.2.6. Wires Thermal Analysis

**Figure 12:**
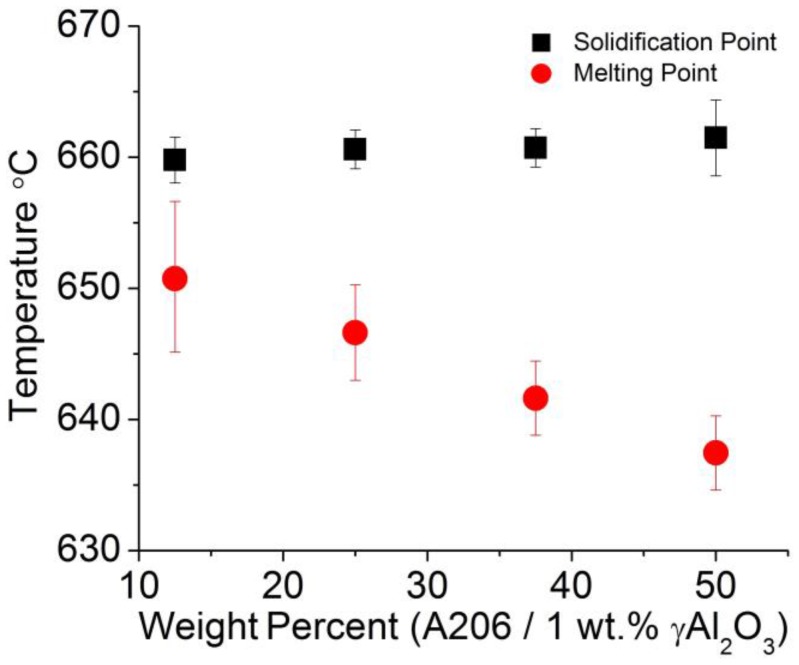
Melting and solidification onset temperatures of aluminum wire samples as measured in a differential thermal analysis apparatus.

**Figure 13:**
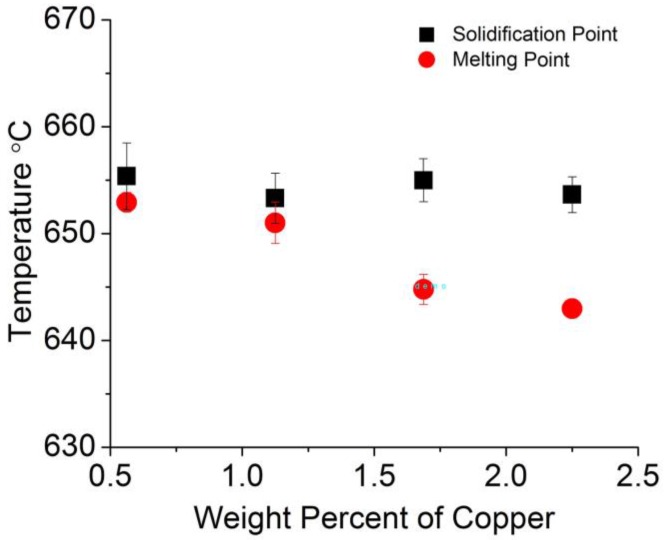
Melting and solidification onset temperatures measured in the Al–Cu wire samples using a differential thermal analyzer.

A differential thermal analysis apparatus allowed determining the onsets of melting (upon heating) and solidification (upon cooling) of the Al-A206/1 wt % γAl_2_O_3_ wire samples. The results are displayed in Figure 12 whereas Figure 13 shows the melting and solidification onsets as a function of the amount of Cu. By combining both graphs, it is apparent that the concentration of copper decreased the melting point of all wire samples, as expected. In effect, this is the natural behavior of aluminum–copper binary alloys in which the addition of copper decreases the liquidus temperature of a given Al–Cu alloy in the aluminum-rich region [27]. In addition, one could observe that the addition of Al_2_O_3_ particles maintained the initial solidification temperature almost constant and decreased the melting onset temperature of the wires by approximately 5 °C. The almost constant solidification onset temperature can be explained by heterogeneous nucleation of aluminum grains when the nanoparticles are likely acting as catalytic substrates for such nucleation events. While aluminum heterogeneous nucleation has been widely studied previously using differential thermal analysis [28,29,30], the use of Al_2_O_3_ particles as nucleation agents was, for instance, discussed by L. Yang [30]. The catalytic potency of the alumina nanoparticles as nucleants was more marked in the Cu-containing alloys. In effect, dissolved copper lowers the liquidus line, which in this case was prevented by the presence of the nanoparticles. Thus, they favored early nucleation events upon cooling.

## 4. Discussion

To study the statistical significance of the effects of both alumina and copper additions (as mentioned, in the form of A206/1 wt % γAl_2_O_3_) on the ultimate tensile strength, electrical conductivity, and melting temperatures of the wires, we carried out a multiple linear regression analysis. The nomenclature used in the equations is as follows: % IACS = percent of International Annealed Copper Standard; T = temperature of the wires in degrees Celsius (measured simultaneously with the electrical conductivity using the four-point probe); UTS = ultimate tensile strength (MPa); % Cu = weight percent of copper, % γAl_2_O_3_ = weight percent of γAl_2_O_3_, and MP = measured melting point.

The analysis of variance (ANOVA) presented in Table 3, Table 4 and Table 5 for the three models provides the ensuing fitted parameters and resulting *p*-values. Equation (1) describes the ultimate tensile strength as a function of the alumina and copper levels; again, the resulting *p*-values for the wt % γAl_2_O_3_ and wt % Cu variables are nil. This indicates that the alumina and copper amounts were very effective in strengthening the wires.

UTS = 70.12 + 30.76·wt % Cu + 595.99·wt % Al_2_O_3_ − 143.89·wt % Cu·wt % Al_2_O_3_ (1)

The regression model in Equation (2) describes how the electrical conductivity of the wires varies as a function of the γAl_2_O_3_ and copper amounts, as well as temperature; the *p*-value is zero for all the parameters. This further corroborates that increasing the alumina and copper lowered the conductivity of the wires. In the case of γAl_2_O_3_, this can be readily explained by the high electric resistivity of alumina at room temperature, which is estimated to exceed 10^12^ Ω·m [22]. The decrease in conductivity of the wire when copper was added is explained by S. Aksöz [31].

% IACS = 64.62 − 26.96·wt % Al_2_O_3_ − 7.46·wt % Cu − 0.13·T+8.84·wt % Cu·wt % Al_2_O_3_ (2)

In Equation (3), the model describes the behavior of the melting point; the *p*-values are zero for wt % γAl_2_O_3_ and wt % Cu, which indicate that alumina and copper decreased significantly the melting points of the wires. The R^2^ values for the resulting regression models (i.e., electrical conductivity, ultimate tensile strength, and melting point) are high: 97.10%, 86.95%, and 94.11%, respectively. This is an important finding if these wires are intended for fillers in TIG welding of aluminum parts: the strength of the welded joint can be improved with the addition of alumina to the filler. At the same time, as the melting point of the wire filler becomes lower, less energy would be required to melt the wire. This advantageous fact is further buttressed by a higher electrical resistivity of the wire (due to the alumina and copper added). In summary, combining both properties, i.e., lower melting points and higher resistivity, one can expect to decrease the energy needed to melt the filler when alumina nanoparticles are present. Nonetheless, one must take into consideration that the content of A206/1 wt % γAl_2_O_3_ must be less than 50% in order to form a sound spool (no cracks) of these novel wires.

MP = 656.22 − 11.32·wt % Al_2_O_3_ − 5.99·wt % Cu (3)

## 5. Conclusions

The experimental results allow several conclusions:

▪ The γAl_2_O_3_ nanoparticles can be successfully added to molten A206 to fabricate an A206/1 wt % γAl_2_O_3_ nanocomposite by semi-solid mixing and ultrasonic processing.
▪ The ultimate tensile strength of the wires can be increased by increasing the amount of γAl_2_O_3_ nanoparticles and Cu added to the aluminum melt.
▪ Increasing the levels of γAl_2_O_3_ nanoparticles and Cu lowers the electrical conductivity and melting point of the wires.
▪ A fractography study revealed that an increment of A206/1 wt % γAl_2_O_3_ nanocomposite in the aluminum matrix leads to more brittleness of the wires.
▪ All those results are also corroborated via statistical analysis. They evince the feasibility of using this new material as a filler in aluminum welding.

## Figures and Tables

**Table 1:** A206 nominal chemical composition [13].

Alloy	wt % Cu	wt % Mn	wt % Mg	wt % Ti
A206.0	4.5	0.3	0.25	0.22

**Table 2:** Wires chemical composition.

Wires	wt % Al	wt % γAl_2_O_3_	wt % Cu	wt % Mn	wt % Mg	wt % Ti
Al-12.5 wt % (A206/1 wt % γAl_2_O_3_)	99.218	0.125	0.562	0.037	0.031	0.027
Al-25.0 wt % (A206/1 wt % γAl_2_O_3_)	98.433	0.250	1.125	0.075	0.062	0.055
Al-37.5 wt % (A206/1 wt % γAl_2_O_3_)	97.651	0.375	1.687	0.112	0.093	0.082
Al-50.0 wt % (A206/1 wt % γAl_2_O_3_)	96.865	0.500	2.250	0.150	0.125	0.110

**Table 3:** Analysis of variance (ANOVA) of the model in Equation (1).

Parameter	Value	Standard Error of the Coefficient	*p*-Value
Constant	70.124	8.319	0.000
wt % Al_2_O_3_	595.989	62.778	0.000
wt % Cu	30.763	5.762	0.000
wt % Cu·wt % Al_2_O_3_	−143.890	32.424	0.001

**Table 4:** ANOVA of the model in Equation (2).

Parameter	Value	Standard Error of the Coefficient	*p*-Value
Constant	64.624	1.387	0.000
wt % Al_2_O_3_	−26.958	9.301	0.000
wt % Cu	−7.456	0.874	0.000
Temperature	−0.125	0.009	0.000
wt % Cu·wt % Al_2_O_3_	8.837	4.787	0.072

**Table 5:** ANOVA of the model in Equation (3).

Parameter	Value	Standard Error of the Coefficient	*p*-Value
Constant	656.221	0.66204	0.000
wt % Al_2_O_3_	−11.315	1.57952	0.000
wt % Cu	−5.987	0.46425	0.000
